# Storage Insects on Yam Chips and Their Traditional Management in Northern Benin

**DOI:** 10.1155/2013/484536

**Published:** 2013-04-04

**Authors:** Y. L. Loko, A. Dansi, M. Tamo, A. H. Bokonon-Ganta, P. Assogba, M. Dansi, R. Vodouhè, A. Akoegninou, A. Sanni

**Affiliations:** ^1^Laboratory of Agricultural Biodiversity and Tropical Plant breeding (LAAPT), Faculty of Sciences and Technology (FAST-Dassa), University of Abomey-Calavi (UAC), P.O. Box 526, Cotonou, Benin; ^2^Crop, Aromatic and Medicinal Plant Biodiversity Research and Development Institute (IRDCAM), 071 BP 28 Cotonou, Benin; ^3^Institut International d'Agriculture Tropicale (IITA), 08 BP 0932 Cotonou, Benin; ^4^Service de la Protection des Végétaux et du Contrôle Phytosanitaire, Direction de l'Agriculture, BP 58 Porto-Novo, Benin; ^5^Bioversity International, Office of West and Central Africa, 08 BP 0932 Cotonou, Benin; ^6^National Herbarium, Department of Botany and Plant Biology, Faculty of Sciences and Technology (FAST), University of Abomey-Calavi (UAC), P.O. Box 526, Cotonou, Benin; ^7^Laboratory of Biochemistry and Molecular Biology, Faculty of Sciences and Technology (FAST), University of Abomey-Calavi (UAC), P.O. Box 526, Cotonou, Benin

## Abstract

Twenty-five villages of Northern Benin were surveyed to identify the constraints of yam chips production, assess the diversity of storage insects on yam chips, and document farmers' perception of their impacts on the stocks and their traditional management practices. Damages due to storage insects (63.9% of responses) and insufficiency of insect-resistant varieties (16.7% of responses) were the major constraints of yam chips production. Twelve insect pest species were identified among which *Dinoderus porcellus* Lesne (Coleoptera, Bostrichidae) was by far the most important and the most distributed (97.44% of the samples). Three predators (*Teretrius nigrescens* Lewis, *Xylocoris flavipes* Reuter, and *Alloeocranum biannulipes* Montrouzier & Signoret) and one parasitoid (*Dinarmus basalis* Rondani) all Coleoptera, Bostrichidae were also identified. The most important traditional practices used to control or prevent insect attack in yam chips were documented and the producers' preference criteria for yam cultivars used to produce chips were identified and prioritized. To further promote the production of yam chips, diversification of insect-resistant yam varieties, conception, and use of health-protective natural insecticides and popularization of modern storage structures were proposed.

## 1. Introduction

In economic terms, yams (*Dioscorea* spp.) are the world's fourth most important tuber crop after potatoes, cassava, and sweet potatoes [1]. They are cultivated in most tropical countries but especially in West Africa, where over 95% of the world's output is produced [1, 2]. They are the main source of carbohydrate for millions of people. In West Africa, many yam species are cultivated but the African domesticates known as the Guinea yams (*D. cayenensis* Lam*.-D. rotundata* Poir. complex) are, however, the most important, most preferred, and widely planted [3]. 

In Benin, the fourth yam-producing country behind Nigeria, Ivory Coast, and Ghana, yam is among the most important food crops [1] and has economic and sociocultural importance [4]. Yam is seasonal and the fresh tubers are highly perishable. Postharvest losses are very high, ranging from 30% to 85% of the total production [4]. In order to overcome this high perishability of the tubers and the irregularity of its availability throughout the year, yams are traditionally processed into dried chips or cossettes [5–7], hence reinforcing food security [7]. Unfortunately, yam chips are often severely attacked by insects, which sometimes reduce whole yam stocks into powder in very few months [8, 9]. Very little research attention has been given to storage insects attack on yam chips and traditional management practices in Benin. Gnonlonfin et al. [10] reported the existence of many species of insects but focused their study mainly on their population's dynamics in stored yams chips. Consequently, the diversity of the insect species in the yam chips producing zone is still unknown, and farmers' perception of the importance of insect damages in the stocks has never been assessed. Traditional management practices (including the storage structures) used to prevent or control insect infestations have also not been documented. Yam chips are produced from tubers of single-harvest varieties, locally known as “Kokoro” characterized by their numerous small-sized tubers. Within Kokoro yams, many varieties of different agronomic and technological characteristics exist [11] but the yam chips producers' variety preference or selection criteria have never been studied. Knowledge of farmers' selection criteria will be useful in designing concrete breeding programmes that could facilitate the adoption of improved varieties [12].

We report in this paper a survey conducted in the most important yam chips producing zone of Benin in order to

- identify and prioritise the constraints related to the production of yam chips in Benin and farmers' propositions for overcoming these constraints,
- document farmers' perceptions about insect pests on stored yam chips and traditional management practices,
- assess the diversity and the importance of the storage insects species in the most important yam chips production zone of Benin,
- Identify and prioritize the producers' variety preference or selection criteria across study zones and ethnic groups for popularization and breeding purposes.

## 2. Material and Methods

### 2.1. Study Area

**Figure 1:**
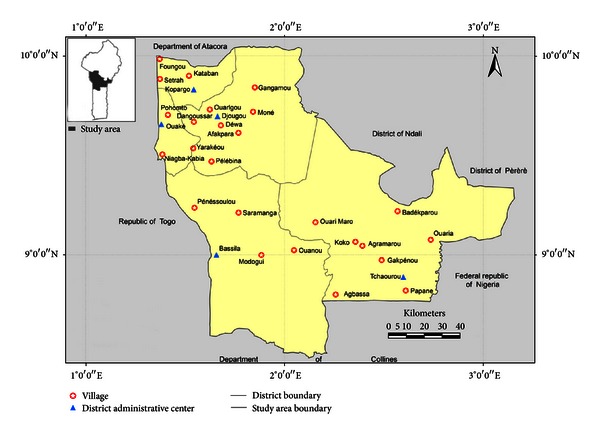
Map of Benin showing the geographical position of the surveyed villages.

**Figure 2:**
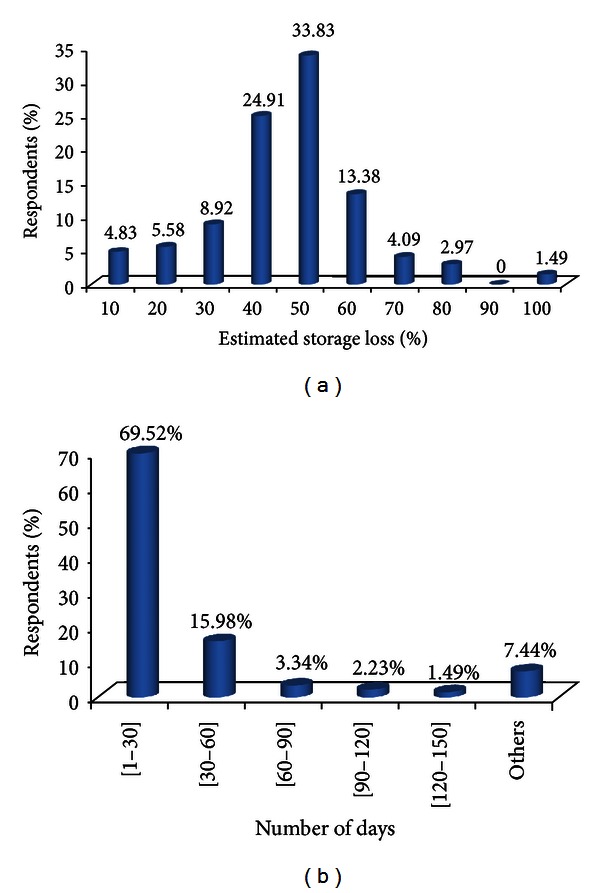
Farmers' perception of (a) storage loss due to stored yam chips insect pests, (b) the period of infestation of the yam chips.

The study was conducted in five districts (Djougou, Copargo, Ouaké, Bassila, and Tchaourou) of the Departments of Donga and Borgou in northern Benin. These districts are known to be the major yam chips production zones of Benin [11, 13]. The inhabitants are members of seven ethnic groups (Figure 1; Table 1) (Ani, Bariba, Lokpa, Nago, Peulh, Taneka, and Yom) and have a very long tradition in processing Kokoro yam tubers into chips or cossettes (Figure 2). The departments of Donga and Borgou are located in a semiarid agroecological zone characterized by unpredictable and irregular rainfall (800–1300 mm/year) with only one rainy season (May to October) and a dry season lasting for more than 5 months sometimes [14]. Mean annual temperatures range from 26°C to 28°C and may exceptionally reach 35°C [15, 16]. Yam production in this area is intensive and essentially based on Kokoro yams [13], which have very variable yields from one season to another due to climatic hazards [13].

### 2.2. Site Selection and Survey

Twenty-five villages (Table 1; Figure 1) were randomly selected throughout the study area and its ethnic zones for the survey. Data were collected from the different sites during expeditions through the application of Participatory Research Appraisal tools and techniques such as granary visits, direct observation, focus group discussions, and individual interviews using a questionnaire and the help of translators from each area following Dansi et al. [12]. In each site, local farmers' associations were involved in the study to facilitate the identification of the households to survey and the data collection. Within villages, 10 to 12 households were randomly selected for individual interviews using the transect method described by Dansi et al. [17]. In each household, the interviewee (head of household or his wife or one of his wives in case of polygamy) was selected by mutual agreement with the hosting couple according to Christinck et al. [18]. Apart from the socioeconomic data such as age, gender, and educational level of the interviewees, data collected included the farmers' perceptions of the constraints of yam chips production, the cossette storage structures and practices, the importance of damages caused by insects, the time of the infestation, the farmers' knowledge of the insect species, the traditional management practices on the infested stocks, and the farmers' preference criteria of kokoro varieties used in the production of the chips. Preference criteria of kokoro varieties were identified and prioritized using the matrix scoring technique described by Defoer et al. [19], Adoukonou-Sagbadja et al. [20], and Dansi et al. [12]. In each village, samples (500 g) of infested yam chips were collected directly from two to three randomly selected yam chips storage structures following Mendesil et al. [21] and Koradaa et al. [22]. Initial weights of the samples to be collected were taken using the numerical balance (model SF-400). Infested samples collected were preserved in plastic containers with perforated lids to allow for ventilation. With the aid of a plant taxonomist at the national herbarium of the University of Abomey-Calavi, insecticides and/or insect repulsive plants used in processing reported by interviewees we sampled and their scientific names were determined using the analytical flora of Benin [16].

### 2.3. Incubation of the Samples and Isolation and Identification of the Insects

The labeled plastic containers containing the samples of the infested chips were incubated for three months under laboratory conditions at temperature of 25–27°C and 70%–80% relative humidity, following the method described by Eze et al. [23]. After the incubation period, the samples were broken into particles of less than 0.5 cm using a hand mortar and the insects were recovered through a 0.25 mm nylon net sieve [24]. Recovered insects were counted and conserved in a flask containing 70% alcohol for safeguarding and identification. Species' identification was done at the Biodiversity Center of the International Institute of Tropical Agriculture (IITA-Benin).

### 2.4. Statistical Analysis

Data were analyzed through descriptive statistics (frequencies, percentages, means, etc.) to generate summaries and tables at different (villages, individuals) levels using SAS software [25].

## 3. Results

### 3.1. Characteristics of the Respondents

The respondents were in majority (98%) women. Sixty-three (63%) are illiterate and 47% attended primary school only. Their ages varied from 17 to 60 years with an average of 37 years. In all the households surveyed, yam chips were produced for either home consumption and for the market (95.2% of the respondents) or for home consumption only (4.8%). 

### 3.2. Constraints of Yam Chips Production

Six constraints (Table 2) related to yam chips production in Benin were recorded. They were all directly or indirectly linked to the storage of the chips. Among them, damages due to storage insects were the most important (63.9% of responses), followed by insufficiency of insect-resistant varieties (16.7% of responses) and the lack of natural human health preserving insecticides (10.2% of responses). The other three constraints (lack of organised markets, low availability of fresh kokoro yam tubers, and the lack of appropriate and specific storage structures) were of very low importance (only 1.1% to 4.5% of responses). The majority of the yam chips producers (72.12%) estimated at 40%–60% the importance of the damage caused by the storage insects on the yam ships (Figure 2(a)). This however depends on the variety used, the conservation structure and the drying level of the chips. For the great majority (92.94%) of the respondents, the infestation of the cossettes in stock occurred during the first two months (Figure 2(b)). In order to minimize these constraints, yam chips producers proposed six key solutions (Table 3) including diversification of good storage insect-resistant kokoro yam (30.2% of responses), development of a natural human health preserving insecticides (24.2% of responses), enhancement of the production of kokoro yam (21.2% of responses), and development of fast drying areas for the yam chips (19.5% of responses).

### 3.3. Farmers' Knowledge of the Insect Pests Damaging Yam Chips in Stocks and Diversity Assessment

**Figure 3:**
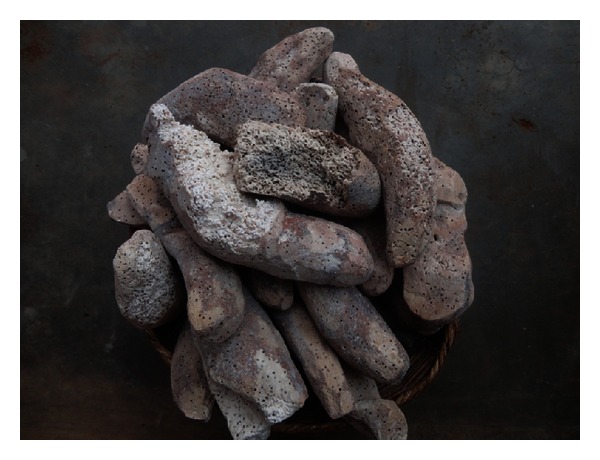
Yam chips with insect infestation in Benin.

In the study area, all the storage insects were traditionally classified in a single group named *Benonkpé* in Ani, *Kokolibo* in Nago, *Doridji* in Peulh, *Gbénénoukokonou* in Bariba, *Dresse* in Yom, and *Poucasse* in Lokpa and Taneka. All these six vernacular names literally mean beetles. Farmers reported that these beetles act by penetrating the chips and drastically reducing their internal parts into powdery waste (Figure 3). Although interviewees recognized storage insects as major constraint in yam chips production, only 47% of them were able to differentiate some species. The few respondents who attempted to identify yam chips insect pests based their identification mainly on the colour (45.12% of responses) and the relative size (32.18% of responses) of the insects and on the symptoms of the damage they caused (22.7% of responses). 

The diversity analysis conducted on the total of 78 samples collected and incubated revealed 12 species of insects belonging to four orders (Table 3) which are Coleoptera (eight species), Hemiptera (two species), Hymenoptera (one species), and Psocoptera (undetermined species). Species of the order of Coleoptera were the most numerous and the most represented in the samples. On average 223 insects of the order Coleoptera were counted by 500 g of yam chips against 11.4 for all the other orders put together (Table 4). Among the species identified *Dinoderus porcellus* Lesne was the most represented. It was found in 97.44% of the collected samples and was also the most abundant in all the samples in which it was found (Table 4). This was followed by the species *Tribolium castaneum* (Herbst), detected in 52.56% of the samples (Table 4). The other species were found in only 2 to 10 samples out of the 78 samples collected and in very few numbers. Among the 12 species of insects identified, three (*Teretrius nigrescens* Lewis, *Xylocoris flavipes* Reuter, and *Alloeocranum biannulipes* Montrouzier & Signoret) were predators and one (*Dinarmus basalis* Rondani) was a parasitoid. *Xylocoris flavipes* was found in 12.82% of the samples and appeared to be the most abundant predator.

### 3.4. Traditional Yam Chips Storage Systems and Duration of the Conservation

In all the households surveyed, yam chips were stored inside houses and rooms in various containers. The great majority (97.77%) of producers used maize bags (made with synthetic materials) of various sizes as containers, depending on the quantity of chips to be conserved. Only few producers (2.23%) preferred to store in large-sized and hermetically closed plastic buckets, jars, or barrels to prevent insect infestations. No specific structure was dedicated to storage of yam chips. Interviewees reported that storage period varied from 1 to 13 months with an average of 8 months. For 49.82%, 31.97%, and 18.21% of the respondents, storage duration of yam chips varied between 1 and 5 months, 5 and 10 months, and 10 and 15 months, respectively. 

### 3.5. Traditional Control Systems of Yam Chips Storage Insects Pests

Under traditional storage conditions, interviewees used seven strategies to reduce losses due to insects (Table 5). Among those, the most important were regular inspection and exposure of chips to sunlight to repel insects (35.93% of responses), use of insect-resistant varieties (26.80% of responses), and use of insecticide and/or insect's repulsive plants during preparation (26.45% of responses). The other strategies such as shaking of the yam chips to remove insects along powdery waste, use of insecticides, treatment with pepper powder, and minimising frequent opening of storage structures to avoid entrance of the insects were poorly used (Table 5). According to interviewees, severely infested stocks of yam chips were sold (66.17% of responses), used for home consumption only (24.54% of responses), or simply thrown away (9.29% of responses). 

The study revealed that eight species of plant were used to prevent infestation of the yam chips or to control insect pests (Table 6). Among these species, three (*Bridelia ferruginea*, *Blighia sapida*, and *Khaya senegalensis*) were reported to be insecticide while four (*Piliostigma thonningii*, *Lophira lanceolata*, *Tectona grandis*, and *Sorghum bicolor*) were said to be dyes and insect repulsive (Table 6). Cassava leaves were also used during the parboiling to harden the chips. *Piliostigma thonningii* and *Sorghum bicolor* were known and used across all the ethnic groups while the other species apart from *Tectona grandis* were each used in only one ethnic group. The number and types of species of plants varied from one ethnic group to another. Yom and Peulh used only two species of plant, Lokpa and Ani used four species, and Nago used five. For the different plant species identified, the plant parts (leaves or bark) used, the application or treatment methods (infusion or fumigation), and the frequencies of utilization across ethnic groups are summarized in Table 6. 

Throughout the study zone, 37 kokoro yam cultivars used to produce chips were listed as tolerant to storage insects. The number of cultivars reported varies across ethnic areas. Eight cultivars were reported with the Nago, Bariba, and Lokpa, seven with the Yom, four with the Taneka, and only two with the Ani (Table 7). In each ethnic area, certain cultivars were more common. With the Nago ethnic group, Oguidigbo, Adakada, Tabané, and Omonya were the most important while with the Bariba ethnic group, the most listed cultivars were Otoukpannan, Tchakatchaka, and Yakanougo (Table 6). 

### 3.6. Farmer's Preferences Criteria for Kokoro Yam Cultivars for Chips Production

Throughout the study zone, kokoro yam cultivars, used for chips production, were selected among the existing diversity based on eight criteria. Among them, the quality of the paste made with the flour, the storage aptitude of the chips, and the quality of the Wassa-Wassa (local couscous made with yam chips' flour) were the most important and represent altogether 74.08% of the responses (Table 8). The number and importance of the criteria also varied across ethnic groups. With the Nago and the Peulh, storage aptitude was the most important criterion while with the other ethnic groups the quality of the paste came at the first position (Table 8). The quality of Wassa-Wassa, which was the third most important criterion among the Lokpa, Ani and Yom people, was not even mentioned by the Bariba people. Similarly, the fast drying quality of the cultivars, which was important to the Nago, the Taneka, and the Ani, was not listed among the Peulh people and had very low values with the other ethnic groups. While all the eight criteria were listed by the Lokpa ethnic group, all but one was recorded with the Taneka and the Yom and only four were identified with the Peulh (Table 8). 

## 4. Discussion

The respondents were in majority women. This can be explained by the fact that in all ethnic groups of the study area, women were the sole processors of kokoro yam tubers into chips. The few males interviewed responded on behalf of their wives, who gave way to them out of respect. The culture of the people was also evident in the yam production system where tasks had been traditionally divided according to gender. Men were in charge of the most important activities in terms of labour requirements, while foods processing and transformation of the yam tubers into chips, among other activities, were devoted to women [4]. According to Bricas and Vernier [26], the commercialization of yam chips is by far more economically profitable than the one of fresh tubers. This could justify the importance that women in the study area gave to commercialization as a means of substantially improving their household income.

Among the constraints related to yam chips production in the study area, damages caused by storage insects stood out as the most important. Similar results were reported by Osuji [27] and Adedire and Gbaye [28] in Nigeria. The importance of the damages raised by the respondents is key indicator of the urgent necessity to develop control strategies against the storage insects. 

Sun drying of infested chips was the major method used by farmers to control these insect pests. This method, which is the oldest known technique of conservation of the agro-alimentary products, also presents several disadvantages. Chalal et al. [29] reported that sun drying directly exposes the products to dust and to ultraviolet rays which can cause the deterioration of food vitamins. Among the solutions proposed by farmers were diversification of good storage insect-resistant kokoro yams and development of natural human health preserving insecticides. These two propositions, which call the attention of plant geneticist and breeders on one hand and industrial chemists on the other, indicate that producers are very concerned about their health. The numerous cases of food poisoning that were associated with the use of cotton insecticides on yam chips recorded these last years in the country and which led to the death of many persons may have contributed to this health consciousness. In Nigeria, Adedoyin et al. [30] and Adeleke [31] also reported poisoning due to the consumption of yam flour (treated with insecticide) in some families in Ilorin and Kano. 

Our study revealed that in the different samples of infested yam chips collected and analyzed, *Dinoderus porcellus* was the most represented. This species which is known to be mostly associated with dried yams [32] has already been reported as the most important pest of stored yam chips in Nigeria [27, 28, 32]. *Dinoderus porcellus* particularly infests well-dried chips [27, 28]. Therefore, it is possible that the few samples, in which it was absent, were not well dried or had relatively higher moisture contents. The presence of *Psocoptera* spp., *Carpophilus dimidiatus*, and *Carpophilus Binotatus* in the samples without *Dinoderus porcellus* supports this hypothesis as they are known as insects associated with wet food products [33]. *Tribolium castaneum* and *Psocoptera *spp. were also found in not negligible number of the samples. The red flour beetle, *Tribolium castaneum*, is a common and one of the most important stored product pests associated with a wide range of durable commodities (barley, bran, cacao, ginger, maize, millet, cassava chips, nutmeg, peanut, pepper, rice, sorghum, etc.) and food-processing facilities worldwide [34–36] were also found. Its presence in the samples examined is not surprising as it has already been reported by Vernier et al. [9], Soldati et al. [37], and Oni and Omoniyi [32]. In some yam chips samples collected outside our study area, Vernier et al. [9, 38] and Gnonlonfin et al. [10] identified five other species which were not found in our studies. These included *Dinoderus bifoveolatus* (Wollaston), *Palorus subdepressus* (Wollaston), *Rhyzopertha dominica* (F.), *C. quadricollis* (Guérin-Méneville), *Gnatocerus maxillosus* (F.), and *Prostephanus truncatus*. In order to have an exhaustive list of all the stored-products insect pests associated with yam chips in Benin and map their geographical distribution, further studies need to be conducted by including the remaining part of the country. A good knowledge of the diversity of the species will be of great utility for the yam breeders who may like to select kokoro cultivars producing tubers that are tolerant to storage insect pests. For example, The Laboratory of Agricultural Biodiversity and Tropical Plant Breeding of the University of Abomey-Calavi (Benin) and the Global Crop Diversity Trust (Rome, Italy) are currently introducing yam chips technology to the arid zone of the department of Atakora (far northwest of Benin), where the environment is quite suitable for fast drying of the chips. To succeed, however, it will be necessary to reckon with kokoro cultivars tolerant to storage insect pests.

To control insect pests and diseases in crops, Integrated Pest Management (IPM) is recommended [39]. IPM promotes biological control based on the use of the natural enemies of pests (predators and parasitoids) and the genetic control through growing of pest and disease tolerant or resistant cultivars [40]. Among the natural enemies encountered in the infested yam chips, *Xylocoris flavipes* is known as an effective polyphagous predator of eggs, larvae and chrysalis of coleopteran insects [41]. This natural enemy is also a predator of larvae of *T. castaneum* [42] and is frequently associated with the insects of cereals stocks [43]. According to Helbig [44], *X. flavipes* has some interesting biological characteristics that make it a potential control agent of storage insect pests. Unfortunately, it was reported that *X. flavipes* only eliminates populations of small-sized insects, but not larger insects or insects with internal feeding such as *D. porcellus* [45]. *A. biannulipes* on the other hand (Montrouzier & Signoret) is known as a predator of the large-sized storage insect pests including *L. serricorne* (F.) and *Tribolium castaneum* [46–48]. A biological control program, combining these predators, will be useful in eliminating various types of insects and will help control the insect pests' complex associated with yam chips.

It appeared from our study that yam chips producers also used diverse plants to protect chips against insect attacks. Phytochemical studies conducted by Dumaine et al. [49] revealed that none of the four plant species (*L. lanceolata*, *T. grandis*, *P. thonningii*, and* B. ferruginea*) used in the study areas has insecticidal or insect repulsing effects. However, Akinpelu and Obuotor [50] found that *P. thonningii* bark extract has a bactericidal activity which is also important for improving the sanitary conditions of the chips. Among the plants used, *Blighia sapida*, *Bridelia ferruginea*, and *Khaya senegalensis* are even believed, and rightly so, to have insecticide properties by the Nago, the Ani, and the Bariba people (Table 6). In fact, the bark extract of *K. senegalensis* has been proved to be antifungal [51], antibacterial [52, 53], and insect antifeedant [54]. Mitchell and Ahmad [55] reported that *B. sapida* has acaricide and insecticide properties. Similarly it has been shown that all the fruit components (skin, aril and granulates, oil) of this plant have repulsive properties against stock insects such as *Callosobruchus maculatus*, *Cryptolestes ferrugineus*, *T. castaneum*, and *S. zeamais* [55–58]. Experiments should be conducted to assess the effects of the extract of these three species on insect pests that damage stored yam chips. 

Chips producers reported that the importance of the damages is a function of the yam cultivars used and listed 37 kokoro yam landraces producing tubers rarely attacked by the storage insect pests. Due to the existence of numerous synonymies in farmer-named yam cultivars [11], these listed landraces may not all correspond to 37 different genotypes. Therefore, agromorphological characterization coupled with molecular analysis should be carried out to identify duplicates and establish the equivalence between recorded names following Tamiru et al. [59] and Kombo et al. [60]. Moreover, and as recommended by Vernier et al. [38], it will be also important to assess by a well-elaborated trial the effectiveness of the tolerance of the chips derived from the tubers of these varieties to storage insect pests. The use of resistant varieties remains the most economically profitable and the best healthy method of combating chips storage insect pests. Because of this, kokoro yams in the chips production zone should be strengthened with more high yielding cultivars that are suitable for chips and resistant to storage insect pests. According to Dansi et al. [11], such cultivars exist in the traditional agriculture and could be identified through participatory evaluation. Within the existing diversity, cultivars to be used for the chips are selected based on diverse criteria, among which those related to the quality of the foods (Wassa-Wassa; paste) made with the yam chips flour and the technological characteristics of the chips are the most important (66.66% of the responses). This result is expected because in Benin, chips are only made and used for food purposes. In the preference criteria identified, aspects related to conservation come in second position indicating that producers really give particular importance to insect damages. The variation of the preference criteria noted across ethnic groups is frequent and has been already reported in many crops such as cowpea [61], banana and plantain [62], maize [63], telf [64], sorghum [65], yam [11], and even fonio [12]. The fast drying criteria importantly raised by the Nago, the Taneka, and the Ani ethnic groups should be seriously considered as it influences the hygienic quality of the chips and their market value. Nago, Taneka, and Ani people mostly produced chips for economic purposes through commercialization. One understands therefore how important fast drying could be to them. 

## 5. Conclusion

This study has allowed us to identify several constraints that hamper yam chips production in northern Benin. Attacks by storage insects were the major constraints identified. Yam chips were infested by various insects, of which the most important was *Dinoderus porcellus*. Several plants are traditionally used to fight these insects. Following farmers' requests, efforts should be directed towards diversification of good kokoro cultivars which are tolerant to storage insects. In this framework and to identify such cultivars, we recommend the participatory evaluation of existing kokoro yam, the identification of duplicates, and clarification of synonymies and the assessment of the tolerance of the chips manufactured with tubers produced by the identified varieties. 

## Figures and Tables

**Table 1:** Administrative localisation of the ethnic areas and sites surveyed.

N°	Ethnic areas	Districts	Number of sites	Selected villages
1	Yom	Djougou	6	Déwa, Alfapara, Pélébina, Mone, Gangamou, Dangoussar
2	Lokpa	Djougou	4	Ouarlgou, Yarakéou, Pohomto, Niagba-kabia
3	Ani	Bassila	2	Penessoulou, Saramanga
4	Nago	Bassila	7	Modogui, Ouanou, Papané, Agramarou, Koko, Agbassa, Wari-Maro
5	Taneka	Copargo	3	Kataban, Setrah, Foungou
6	Bariba	Tchaourou	2	Woria, Badékparou
7	Peulh	Tchaourou	1	Gakpenou

**Table 2:** Yam chips production constraints in Benin.

Constraints	Percentage of responses
Damages caused by storage insects	63.9
Insufficiency of insect-resistant varieties	16.7
Lack of natural human health preserving insecticides	10.2
Lack of appropriate and specific storage structures	4.5
Insufficient availability of fresh kokoro yam tubers	3.6
Lack of organised markets	1.1

**Table 3:** Solutions for the constraints and their importance as proposed by the interviewees in the study area.

Solutions	Importance (% of responses)
Diversification of good storage insect-resistant kokoro yam	30.2
Development of a natural human health preserving insecticides	24.2
Enhancement of the production of kokoro yam	21.2
Development of fast drying areas for the yam chips	19.5
Development of efficient and specific yam chips storage structures	3.3
Establishment of a well-organised yam chips good market	1.6

**Table 4:** Results of the samples incubated at the laboratory showing the species of insects used and their relative abundance.

Types of insects	Infested samples (%)	Average count for 500 g	Percentage of abundance (500 g)	Rank
*Dinoderus porcellus *	76 (97.44)	208.72	89.03	1
*Psocoptera *spp.	19 (24.36)	8.83	3.77	2
*Tribolium castaneum *	41 (52.56)	6.12	2.61	3
*Lasioderma serricorne *	4 (5.13)	3.3	1.41	4
*Sitophilus zeamais *	10 (12.82)	2.57	1.09	5
*Xylocoris flavipes *	10 (12.82)	2.46	1.05	6
*Cryptolestes pusillus *	10 (12.82)	1.26	0.54	7
*Carpophilus dimidiatus *	7 (8.97)	0.85	0.36	8
*Teretrius nigrescens *	5 (6.41)	0.13	0.05	9
*Carpophilus binotatus *	3 (3.85)	0.09	0.04	10
*Alloeocranum biannulipes *	5 (6.41)	0.09	0.04	11
*Dinarmus basalis *	2 (2.56)	0.03	0.01	12

**Table 5:** Farmers' management practices for the control of yam chips insect pests.

Management practices	Importance (% of responses)
Exposure of the infested yam chips to sun	35.93
Use of insect-resistant varieties	26.80
Use of insecticide and/or insect's repulsive plants during preparation	26.45
Sifting of yam chips to remove insects along powdery waste	8.59
Use of insecticides	1.12
Treatment with pepper powder	0.74
Minimising frequent opening of storage structures to avoid entrance of the insects	0.37

**Table 6:** List of plants used to protect yam chips against storage insect pests and their utilisation methods.

Species	Part used	Role	Method of application	Percentage of farmers using the plants across ethnic groups						
				Peulh	Nago	Ani	Taneka	Bariba	Lokpa	Yom
*Piliostigma thonningii *	Leaf/bark	Dye	Infusion	42.86	19.40	15	30.30	16.67	26.47	30.30
*Lophira lanceolata *	Leaf	Dye	Infusion	—	7.46	—	—	—	—	—
*Blighia sapida *	Leaf	Insecticide	Infusion	—	5.97	—	—	—	—	—
*Bridelia ferruginea *	Leaf/bark	Insecticide	Infusion	—	—	5	—	—	—	—
*Khaya senegalensis *	Bark	Insecticide	Fumigate	—	—	—	—	11.11	—	—
*Tectona grandis *	Leaf	Dye	Infusion	—	19.40	5	12.12	—	14.71	—
*Manihot esculentus *	Leaf	Hardening of the yam chips	Infusion	—	—	—	—	—	2.94	—
*Sorghum bicolor *	Stem/oil cakes	Dye	Infusion	57.14	47.77	75	57.58	72.22	55.88	69.70

**Table 7:** Kokoro yam cultivars tolerant to storage insect pests and their importance across ethnic areas.

Ethnic areas	Insect-resistant varieties	Importance (number of farmers)
Ani	Demkpenai	14
	Awanawou	11

	Otoukpannan	18
	Tchakatchaka	16
	Yakanougo	15
Bariba	Omonya	8
	Singor	6
	Ankakorouwoura	1
	Gaboubaba	1
	Kourakourogouroko	1

	Azowi	17
	Iootchra	14
	Moghoun	12
Lokpa	Kounto	10
	Kparokoumè	8
	Soprova	6
	Tougbana	4
	Tédoman	1

	Oguidigbo	54
	Adakada	45
	Tabané	41
Nago	Hounbonon	33
	Kokorogbambe	26
	Kokorolakolako	18
	Kokoroagbalè	13
	Adjawoungbo	5

	Atawouraï	27
Taneka	Souwoukou	19
	Gréé	7
	Djèssoumè	4

	Koutonouman	51
	Biboï	44
Yom	Assinakpeina	39
	Adjôgba	17
	Mouhame	3
	Ayè	2
	Satchila	2

**Table 8:** Famer's preference criteria of good kokoro yam cultivar for chips production in the study area and across ethnic groups.

Preferences criteria	Study area (% of responses)	Ethnic groups (% of responses)						
		Nago	Peulh	Bariba	Taneka	Lokpa	Yom	Ani
Quality of the paste	35.62	23.03	21.43	41.38	38.15	31.25	35.65	32.14
Storage aptitude of the chips	26.5	31.99	57.14	37.93	24.5	23.96	22.61	28.57
Quality of Wassa-Wassa	11.96	4.11	7.14	0	3.54	22.92	13.04	17.86
Colour of the paste	9.67	12.33	14.29	13.79	2.7	6.25	13.04	3.57
Flour richness of the yam chips	3.05	2.74	—	—	7.9	7.29	0	—
Crushing facility of the chips	1.02	—	—	—	2.6	2.08	0.87	—
Taste of the paste	4.83	—	—	3.45	—	5.21	10.44	3.57
Fast drying	7.35	25.8	—	3.45	20.61	1.04	4.35	14.29
